# Effect of Perioperative Oral Management on Hospital Stay in Patients Undergoing Hematopoietic Stem Cell Transplantation: A Retrospective Case-Control Study

**DOI:** 10.7759/cureus.91621

**Published:** 2025-09-04

**Authors:** Yura Iijima, Makoto Yoshimitsu, Takatoshi Hiroshimaya, Takashi Nishiyama, Marika Marutani, Kei Fujishima, Kenji Ishitsuka, Naofumi Tamaki

**Affiliations:** 1 Department of Preventive Dentistry, Kagoshima University Graduate School of Medical and Dental Sciences, Kagoshima, JPN; 2 Department of Hematology and Rheumatology, Kagoshima University Graduate School of Medical and Dental Sciences, Kagoshima, JPN

**Keywords:** hematologic cancer, hematopoietic stem cell transplantation, hospital stay, oral hygiene, perioperative oral management

## Abstract

Background

Prolonged hospitalization in patients with hematologic and collagen diseases presents significant challenges for patient recovery and healthcare resource utilization. Perioperative oral management (POM) has been suggested to reduce systemic complications; however, its impact on length of hospital stay remains unclear.

Methods

This retrospective case-control study analyzed 278 patients admitted to the Department of Hematology and Rheumatology at Kagoshima University Hospital between November 2009 and January 2025. Patients who received POM during hospitalization were compared with those who did not. Prolonged hospitalization was defined as a length of stay in the top 25% of the overall distribution. Multivariable logistic regression analyses were conducted to evaluate the association between POM and prolonged hospitalization, adjusting for age, sex, BMI, smoking status, diabetes mellitus, transplantation type, and disease categories. To minimize selection bias and confounding, inverse probability of treatment weighting (IPTW) based on propensity scores was applied.

Results

Of the 278 patients, 184 received POM. The absence of POM was significantly associated with prolonged hospitalization in both unadjusted and multivariable-adjusted analyses (odds ratio [OR]: 3.70, *p* < 0.001). After applying IPTW, covariate balance between the POM and non-POM groups was adequately achieved (standardized differences < 0.1 for all covariates). Logistic regression analysis after IPTW confirmed that the absence of POM remained significantly associated with prolonged hospitalization (OR: 1.22, *p* < 0.001), demonstrating the robustness of this association.

Conclusion

Perioperative oral management during hospitalization was independently associated with a reduced risk of prolonged hospitalization in patients with hematologic diseases. These findings suggest that incorporating POM into inpatient care may contribute to improved clinical outcomes and more efficient utilization of hospital resources. Further prospective studies are warranted to validate these findings.

## Introduction

Patients with hematologic and collagen vascular diseases often require prolonged hospitalization due to the complexity of their treatment regimens, which include immunosuppressive therapy, hematopoietic stem cell transplantation (HSCT), and the management of infectious complications [1, 2]. Extended hospital stays are associated with increased risks of nosocomial infections, decreased functional capacity, nutritional decline, and substantial healthcare costs [3-6].

Oral health plays a pivotal role in maintaining systemic health, particularly in immunocompromised individuals. Poor oral hygiene has been linked to a higher incidence of systemic inflammation, aspiration pneumonia, and bacteremia, which may further compromise treatment outcomes in hospitalized patients [7-10]. Among patients undergoing chemotherapy or transplantation, oral complications such as mucositis and periodontitis can result in pain, delayed treatment, poor nutritional intake, and an increased risk of secondary infections [11]. Given these risks, proactive oral health management has been emphasized as an essential component of supportive care in hospitalized patients.

Perioperative oral management (POM) is a comprehensive intervention aimed at maintaining and optimizing oral hygiene and function throughout the perioperative period. POM primarily seeks to prevent postoperative complications such as hospital-acquired pneumonia and surgical site infections, as well as treatment-related adverse events like oral mucositis (OM). Unlike routine oral care, POM is systematically integrated into perioperative care pathways through multidisciplinary collaboration between medical and dental professionals [12, 13].

Previous studies have demonstrated that POM interventions, including mechanical plaque removal and mucosal management, can significantly reduce the incidence of hospital-acquired pneumonia and other systemic infections, thereby improving overall clinical outcomes [9, 14-16]. In allogeneic HSCT patients, perioperative oral care interventions have been associated with reduced systemic antibiotic usage and shorter hospital stays, as demonstrated in a study utilizing the Diagnosis Procedure Combination database [17].

However, existing evidence predominantly focuses on specific subgroups, such as elderly inpatients or allogeneic HSCT recipients, leaving the broader applicability of oral functional management across various transplantation types and disease categories underexplored [9, 14-17]. Moreover, the clinical impact of individualized, multidisciplinary oral care interventions tailored to the mucosal and hematologic conditions of patients with hematologic and collagen vascular diseases remains insufficiently clarified.

Therefore, in this study, we aimed to evaluate the clinical significance of POM in patients with hematologic diseases by conducting a detailed analysis using real-world clinical data from Kagoshima University Hospital, while taking into account individual patient factors and intervention specifics.

## Materials and methods

Study design and setting

This retrospective observational study was conducted at Kagoshima University Hospital. Medical records of patients admitted to the Department of Hematology and Rheumatology between November 2009 and January 2025 were reviewed.

Participants

A total of 278 patients diagnosed with hematologic diseases were included in this study. Patients were classified into two groups according to whether they received POM during hospitalization. Transplantation types were categorized as allogeneic bone marrow transplantation (allo-BMT), allogeneic peripheral blood stem cell transplantation (allo-PBSCT), autologous peripheral blood stem cell transplantation (auto-PBSCT), and umbilical cord blood transplantation (UCBT).

Perioperative oral management protocol

POM interventions were implemented by a multidisciplinary team comprising dentists, dental hygienists, and medical staff. The protocol was flexibly adapted to each patient’s oral mucosal condition, hematologic status, and overall treatment plan. The key components of the POM protocol included:

Plaque and Calculus Removal

Supragingival scaling was performed using ultrasonic scalers to remove calculus deposits. Professional mechanical tooth cleaning was carried out utilizing rubber cups, polishing cones, brushes, and contra-angle handpieces to remove dental plaque and polish tooth surfaces. For patients unable to tolerate conventional instrumentation (e.g., due to trismus or severe oral mucositis), mechanical cleaning was performed using sponge brushes and oral moisturizing gels to gently manage biofilm and debris.

Mucosal Care and Functional Exercises

Stimulation and mobility exercises of the buccal mucosa were performed using sponge brushes, accompanied by salivary gland massage targeting the parotid, submandibular, and sublingual glands to promote salivary flow. Gentle oral functional exercises, including cheek and lip movements, were implemented to maintain oral muscle tone and function.

Oral Hygiene Instruction

Patients received individualized oral hygiene instruction tailored to their oral mucosal condition. Appropriate cleaning tools, including standard toothbrushes, soft-bristled toothbrushes, sponge brushes, and one-tuft brushes, were selected according to each patient’s specific needs. For denture wearers, ultrasonic cleaning with detergents containing sodium hypochlorite was performed, and individualized guidance on denture cleaning methods was provided. Gargling practices were also instructed based on the patient's mucosal condition. Oral hygiene methods and tools were dynamically modified in response to changes in mucosal and hematologic status, such as neutropenia and thrombocytopenia. Adherence to oral hygiene instructions was assessed at each clinical visit, typically once every one to two weeks, through oral examination and patient interviews. Additional guidance was provided as needed to reinforce compliance.

Comprehensive Oral Mucosal Care

Comprehensive mucosal care was provided to manage OM and other mucosal conditions according to severity. Azunol® (azulene) 4% (ROHTO NITTEN Co., Ltd., Aichi, Japan) mouthwash was routinely used for mucosal conditioning, while a compounded Azunol®-Xylocaine® rinse (Sandoz Pharma K.K., Tokyo, Japan) was prescribed for patients experiencing severe pain. Episil® (Meiji Seika Pharma Co., Ltd., Tokyo, Japan) oral liquid was applied as a protective barrier for extensive mucosal lesions. Oral moisturizing gels were used to alleviate dryness and to debride necrotic tissue or tongue coatings when necessary. Gargling instructions were provided to prevent infection and reduce mucosal inflammation, with rinsing solutions and frequencies adjusted based on clinical findings.

Nutritional and Feeding Management in Collaboration with the Nutrition Support Team (NST)

The oral care team collaborated with the NST to assess patients' oral function and to provide feedback on appropriate nutritional intake strategies. For patients with OM or dysphagia, coordinated efforts with the NST and rehabilitation staff were undertaken to modify feeding plans, including dietary adjustments and swallowing rehabilitation, in order to support systemic recovery and ensure safe oral intake.

Data collection and outcomes

Demographic and clinical data, including age, sex, body mass index (BMI), body temperature ≥ 37.5℃, smoking status, diabetes mellitus, hypertension, and hyperlipidemia, as well as transplantation type, disease categories, and length of hospital stay, were collected from medical records. Disease categories included malignant lymphoma, leukemia/myelodysplastic syndromes (MDS), multiple myeloma, and others (POEMS syndrome, aplastic anemia, malignant soft tissue tumor of the right thigh, Langerhans cell histiocytosis, and primary systemic AL amyloidosis). The primary outcome was prolonged hospitalization, defined as a hospital stay exceeding the upper quartile of the study cohort's length of stay distribution.

Statistical analysis

Prolonged hospitalization was defined as a length of stay within the top 25% of all patients. Patients were categorized into two groups: those with prolonged hospitalization and those without. Demographic and clinical characteristics were compared between the two groups, and medians, interquartile ranges, and *p*-values were calculated. The *χ*2-test was used to analyze categorical variables, and the Mann-Whitney *U* test was applied to continuous variables.

Logistic regression analysis was performed to evaluate the association between POM and prolonged hospitalization, adjusting for potential confounders including age, sex, BMI, smoking status, diabetes, transplantation type and disease categories.

Because of the small number of patients in the non-POM group, there was a concern of overfitting in the multivariable regression analysis. Therefore, inverse probability of treatment weighting (IPTW) based on propensity scores was applied to minimize selection bias and increase the effective sample size of the control group. Covariate balance after weighting was assessed using standardized differences.

All statistical analyses were performed using Stata version 17 (StataCorp LLC, College Station, TX), and a two-sided *p*-value of <0.05 was considered statistically significant.

Ethics and registration

This study was conducted in accordance with the Declaration of Helsinki. Ethical approval was obtained from the institutional review board of Kagoshima University (approval number: 240041). The data used in this study were derived from pre-existing pseudonymized clinical records that had been processed in such a way that individual patients could not be identified.

## Results

Patient characteristics

Patient demographic and clinical characteristics are summarized in Table 1. There were no significant differences in age, sex, BMI, smoking status, diabetes mellitus, transplantation type, or disease categories between patients with prolonged hospitalization and those without.

**Table 1:** Characteristics of the patients according to hospital stay BMT: bone marrow transplantation, allo-BMT: allogeneic bone marrow transplantation, allo-PBSCT: allogeneic peripheral blood stem cell transplantation, auto-PBSCT: auto-peripheral blood stem cell transplantation, UCBT: umbilical cord blood transplantation

Demographic characteristics		Hospital stay				p	z	
		Others (n=209)		Long stay (n=69)				
		Median	IQR	Median	IQR			
Age		55	43-62	53	43-59.5	0.206	1.266	
BMI		21.7	19.1-24.3	20.8	19.1-23.1	0.133	1.502	
Body temperature	day	8	4-13	15.5	4-13	<0.001	-5.042	
≥37.5℃								
Clinical characteristics		n	%	n	%	p	χ²	
Sex	Male	118	56.5	37	53.6	0.681	0.169	
Smoking	Yes	69	33	28	40.6	0.253	1.307	
Perioperative oral management	Yes	149	81	35	19	0.002	9.805	
Diabetes mellitus	Yes	5	2.4	1	1.5	0.640	0.219	
Hypertension	Yes	3	1.4	1	1.5	0.993	0.000	
Hyperlipidemia	Yes	0	0	0	0	–	–	
Transplantation type	allo-BMT	44	21.1	24	34.8	<0.001	42.520	
	allo-PBSCT	23	11	24	34.8			
	auto-PBSCT	119	56.9	10	14.5			
	UCBT	23	11	11	12.2			
Diseases categories	Malignant lymphoma	68	32.5	37	53.6	<0.001	24.560	
	Leukemia/myelodysplastic syndrome	64	30.6	27	39.1			
	Multiple myeloma	65	31.1	2	2.9			
	Others	12	5.7	3	4.4			

Length of stay and prolonged hospitalization

Prolonged hospitalization was defined as a length of stay within the top 25% of the overall distribution. Among the 278 patients, 69 (24.8%) were classified into the prolonged hospitalization group. Of these, 35 patients (50.7%) had received POM, whereas 149 patients (71.3%) in the short-stay group had received POM. The proportion of patients who received POM was significantly higher in the short-stay group compared to the prolonged hospitalization group.

Logistic regression analysis

The results of logistic regression analyses are presented in Table 2. In the univariate analysis (Model 1), the absence of perioperative oral management (POM) was significantly associated with prolonged hospitalization (odds ratio [OR]: 2.41, *p* = 0.002). This association remained significant in the multivariable logistic regression model adjusting for age and sex (Model 2) (OR: 2.31, *p* = 0.004). Further adjustment for BMI, smoking status, diabetes mellitus, transplantation type, and disease categories (Model 3) also demonstrated a significant association between the absence of POM and prolonged hospitalization (OR: 3.70, *p* < 0.001).

**Table 2:** Logistic regression analysis of variables associated with hospital stay Model 1: univariate analysis, Model 2: adjusted for age and sex, Model 3: Model 2 + adjusted for smoking, diabetes mellitus, transplantation type and disease categories ORs: odds ratio, CI: confidence interval, BMT: bone marrow transplantation, allo-BMT: allogeneic bone marrow transplantation, allo-PBSCT: allogeneic peripheral blood stem cell transplantation, auto-PBSCT: auto-peripheral blood stem cell transplantation, UCBT: umbilical cord blood transplantation

Variables	ref		Model 1				Model 2				Model 3			
			OR	95% CI	p	z	ORs	95% CI	p	z	ORs	95% CI	p	z
POM	(+)	(-)	2.41	1.38-4.22	0.002	3.09	2.31	1.32-4.06	0.004	3.02	3.70	1.81-7.57	<0.001	3.54
Age	<60	≥60	–	–	–		1.70	0.91-3.19	0.095	0.48	1.10	0.52-2.31	0.804	0.49
Sex	female	male	–	–	–		1.12	0.64-1.97	0.681	0.36	1.28	0.60-2.72	0.517	0.77
Smoking	No	Yes	–	–	–		–	–	–		1.71	0.78-3.71	0.178	1.47
Diabetes mellitus	No	Yes	–	–	–		–	–	–		5.00	0.47-53.5	0.183	-1.35
Transplantation type	allo-PBSCT	allo-BMT	–	–	–		–	–	–		6.39	2.31-17.66	<0.001	1.75
		auto-PBSCT	–	–	–		–	–	–		13.9	4.61-41.89	<0.001	6.04
		UCBT	–	–	–		–	–	–		9.56	2.82-32.44	<0.001	0.47
Diseases categories	Leukemia	Malignant lymphoma	–	–	–		–	–	–		2.72	1.30-5.69	0.008	-2.58
	Multiple myeloma	Malignant lymphoma	–	–	–		–	–	–		5.21	1.01-26.69	0.048	0.28

To address potential confounding factors and mitigate the risk of overfitting, IPTW based on propensity scores was applied. After weighting, standardized differences for all covariates were reduced to less than 0.1, indicating adequate balance between the POM and non-POM groups (Table 3). Logistic regression analysis after IPTW adjustment confirmed that the absence of POM remained significantly associated with prolonged hospitalization (OR: 1.23, *p* < 0.001), supporting the robustness of this association.

**Table 3:** Patient characteristics stratified by POM with propensity score-based IPTW adjustment CI: confidence interval, POM: perioperative oral management, IPTW: inverse probability of treatment weighting

Variables	Generalized linear analysis after IPTW		
	Odds ratio (95% CI)	p	z
POM	1.23 (1.11–1.36)	<0.001	3.91

## Discussion

Main findings and interpretation

In this retrospective observational study, we demonstrated that inpatient oral functional management was independently associated with a reduced risk of prolonged hospitalization among patients with hematologic and collagen vascular diseases. Patients who received oral care interventions had significantly lower odds of experiencing extended hospital stays, even after adjusting for potential confounding factors such as age, sex, BMI, smoking status, diabetes mellitus, transplantation type, and disease categories.

Our comprehensive oral care protocol, which was flexibly tailored to each patient’s mucosal condition and hematologic status, facilitated safe and effective interventions even in high-risk cases, such as those with severe mucositis or cytopenia. This individualized approach likely contributed not only to infection control but also to the maintenance of oral intake, prevention of functional decline, and promotion of overall recovery.

Notably, the association between oral care interventions and shortened hospital stays was consistently observed across various transplantation procedures, including autologous and cord blood transplantation, as well as across diverse disease categories such as malignant lymphoma, leukemia/MDS, and multiple myeloma. These findings suggest that the clinical benefits of inpatient oral care interventions may extend beyond specific procedural or demographic subgroups, underscoring their broader applicability in real-world clinical practice.

Comparison with previous studies

Several previous studies have demonstrated that oral care interventions in hospitalized patients contribute significantly to infection control and the reduction of hospital stay duration [16-18]. Yoneyama et al. reported that professional oral care reduced the incidence of aspiration pneumonia in elderly inpatients, highlighting the systemic impact of oral hygiene management in vulnerable populations [18]. Building upon this, Kubo et al. conducted a systematic review and meta-analysis in hospitalized cancer patients, demonstrating that perioperative oral care significantly reduced the incidence of postoperative pneumonia and surgical site infections, while also contributing to shorter hospital stays [16]. In the field of hematopoietic stem cell transplantation (HSCT), Moriwaki et al. performed a multicenter retrospective study and reported that perioperative oral care in allogeneic transplant recipients led to a significant reduction in systemic antibiotic use and hospitalization duration, suggesting that oral care interventions contribute to improved systemic outcomes even under immunosuppressive conditions [17]. Collectively, these findings underscore the role of oral care as an essential component not only for local hygiene maintenance but also for systemic infection control and facilitation of recovery across diverse patient populations.

While previous studies have provided robust evidence supporting the clinical benefits of oral care interventions, they have predominantly focused on specific patient subgroups, such as elderly inpatients or allogeneic transplant recipients [17, 18]. In contrast, our study demonstrated that individualized oral functional management was associated with a significant reduction in prolonged hospitalization across a broader range of clinical contexts. This included not only allogeneic but also autologous and cord blood transplant recipients, as well as diverse disease categories such as malignant lymphoma, leukemia/ MDS, and multiple myeloma.

These findings underscore the versatility and broader applicability of a patient-centered, multidisciplinary oral care protocol across a wide range of immunocompromised populations. Our results suggest that tailored oral functional management can be effectively integrated into routine inpatient care pathways, thereby extending its clinical impact beyond the traditionally studied high-risk groups.

Potential mechanisms

Several mechanisms could explain the observed reduction in hospitalization duration associated with POM. First, professional oral care reduces oral microbial load and improves oral hygiene, thereby lowering the risk of systemic infections such as bacteremia and hospital-acquired pneumonia [19, 20]. Second, maintaining oral function through oral hygiene instruction and functional exercises may help prevent the onset of OM and support adequate oral intake. OM commonly develops as an adverse effect of conditioning regimens in patients undergoing hematopoietic stem cell transplantation [21, 22]. It is often accompanied by severe pain and extensive ulcerations, which make oral ingestion difficult and severely restrict nutritional intake. OM has been reported to be one of the most debilitating side effects experienced by patients undergoing bone marrow transplantation [23-25]. Therefore, preventing the development of OM may help preserve nutritional status, promote faster recovery, and facilitate early mobilization and discharge readiness. Third, preserving oral function enhances patients' ability to chew, swallow, and communicate, which in turn promotes adequate nutritional intake, prevents aspiration, and supports more effective rehabilitation. These improvements may contribute to the maintenance of activities of daily living and quality of life, thereby enabling early mobilization and readiness for discharge [26-28].

Additionally, improved oral health status has been associated with reductions in systemic inflammatory markers, such as C-reactive protein (CRP) and interleukin-6 (IL‑6) [29]. The reduction of systemic inflammation through periodontal treatments, including scaling and debridement, may contribute to clinical stabilization and expedite discharge readiness.

Furthermore, the structured POM protocol itself likely contributed to the positive outcomes by providing a comprehensive and multidisciplinary framework for oral care delivery [18, 19]. The integration of systematic plaque control, individualized mucosal management, functional rehabilitation, and collaborative care coordination ensured that patients received consistent and proactive oral care throughout their hospitalization. This proactive management approach likely minimized the incidence of oral complications, reduced infection risks, and facilitated early recovery, thereby contributing to the reduction in hospital stay duration.

Clinical implications

Our findings suggest that integrating inpatient POM into routine care pathways can contribute not only to infection prevention but also to the reduction of hospital stay duration in patients with hematologic and collagen vascular diseases. Given the increasing demand for efficient resource utilization and shorter hospitalization periods, POM interventions offer a practical solution that is both cost-effective and high-impact.

Okubo et al. reported that professional oral care interventions for nursing home residents effectively prevented pneumonia, demonstrating a favorable incremental cost-effectiveness ratio of approximately JPY 4,000,000 per quality-adjusted life year [30]. This evidence underscores the economic value of structured oral care protocols, highlighting their potential to improve patient outcomes while simultaneously alleviating the burden on healthcare systems.

Implementing comprehensive oral care frameworks and establishing coordinated dental referral processes could strengthen collaborative healthcare delivery, thereby contributing to improved patient outcomes and healthcare system efficiency.

Limitations

This study has several limitations. First, it was a single-center retrospective observational study, which may limit the generalizability of the findings. The data reflect the clinical practices and patient population of Kagoshima University Hospital and may not fully represent other healthcare settings with differing institutional protocols or patient demographics. Second, although adjustments were made for several confounding factors, including age, sex, BMI, smoking status, and diabetes mellitus, there may have been unmeasured variables, such as disease severity or functional status, that could have influenced hospitalization length. Despite the use of IPTW based on propensity scores to reduce selection bias and confounding, this method can only adjust for measured covariates, and residual confounding due to unmeasured factors cannot be entirely excluded. Third, the decision to refer patients for oral management was not randomized and may have been influenced by clinical judgement, introducing selection bias. Although IPTW analysis was employed to emulate the conditions of a randomized controlled trial by statistically balancing baseline characteristics, the possibility of referral bias remains inherent in observational study designs. Fourth, this study did not include an a priori sample size calculation; instead, all consecutive patients admitted during the study period were analyzed. Fifth, the extended 15-year study period raises the possibility that advances in medical technology, supportive care, and changes in hospitalization systems or discharge criteria could have influenced the outcomes. While statistical adjustments were performed, the impact of such time-dependent factors cannot be fully excluded. Sixth, although the general framework of POM remained consistent throughout the study, the timing and intensity of interventions were tailored to each patient’s condition, potentially introducing heterogeneity in its implementation. Finally, the frequency of POM referrals over time was not quantified, and therefore changes in referral patterns across the study period may have introduced additional selection bias. Future prospective multicenter studies with larger cohorts are warranted to validate these findings and further clarify the mechanisms underlying the observed benefits of POM.

Future directions

Prospective studies, including randomized controlled trials, are needed to confirm the causal relationship between inpatient POM and hospitalization outcomes. Additionally, further research should investigate the cost-effectiveness of routine oral care interventions across diverse healthcare settings and elucidate the specific biological and functional mechanisms through which oral care contributes to recovery in patients with hematologic diseases.

Furthermore, studies focusing on the development and validation of standardized POM protocols, as well as their scalability and implementation feasibility across different institutions, will be essential to establish evidence-based guidelines for oral management in immunocompromised populations.

## Conclusions

This retrospective observational study demonstrated that POM was significantly associated with a reduced risk of prolonged hospitalization among patients with hematologic diseases. Even after adjusting for multiple confounding factors and applying IPTW to minimize selection bias, the association between POM and shorter hospital stays remained robust. These findings suggest that integrating POM into the inpatient care of patients with hematologic disease may contribute to more efficient recovery and optimized utilization of hospital resources. Further prospective multicenter studies are warranted to validate these findings and elucidate the mechanisms underlying the clinical benefits of POM in this patient population.

## References

[1] Dykewicz CA (2001). Summary of the guidelines for preventing opportunistic infections among hematopoietic stem cell transplant recipients. Clin Infect Dis.

[2] Chen CY, Tsay W, Tang JL, Tien HF, Chen YC, Chang SC, Hsueh PR (2010). Epidemiology of bloodstream infections in patients with haematological malignancies with and without neutropenia. Epidemiol Infect.

[3] Bo M, Fonte G, Pivaro F (2016). Prevalence of and factors associated with prolonged length of stay in older hospitalized medical patients. Geriatr Gerontol Int.

[4] Barba R, Marco J, Canora J (2015). Prolonged length of stay in hospitalized internal medicine patients. Eur J Intern Med.

[5] Kato N, Kondo M, Okubo I, Hasegawa T (2014). Length of hospital stay in Japan 1971-2008: hospital ownership and cost-containment policies. Health Policy.

[6] Liu H, Zhao J, Xing Y, Li M, Du M, Suo J, Liu Y (2014). Nosocomial infection in adult admissions with hematological malignancies originating from different lineages: a prospective observational study. PLoS One.

[7] Varoni EM, Rimondini L (2022). Oral microbiome, oral health and systemic health: a multidirectional link. Biomedicines.

[8] Peng X, Cheng L, You Y (2022). Oral microbiota in human systematic diseases. Int J Oral Sci.

[9] Yoneyama T, Yoshida M, Ohrui T (2002). Oral care reduces pneumonia in older patients in nursing homes. J Am Geriatr Soc.

[10] Natarajan P, Madanian S, Marshall S (2025). Investigating the link between oral health conditions and systemic diseases: a cross-sectional analysis. Sci Rep.

[11] Arduino PG, Gambino A, Giaccone L (2022). Oral health status after hematopoietic stem cell transplantations: outcomes from an adult Italian population. Spec Care Dentist.

[12] Shimane T, Koike K, Fujita S (2023). Positive impact of perioperative oral management on the risk of surgical site infections after abdominal surgery: sixteen universities in Japan. Medicine (Baltimore).

[13] Camus-Jansson F, Longueira-Diaz N, Salinas-Diaz B, Granic-Chinchón A, Cueto-Urbina W, Parra-Parra M, Lopez-de-Blanc SA (2023). Preoperative oral practices and incidence of postoperative complications in hospital medical-surgical procedures: a meta-analysis. Med Oral Patol Oral Cir Bucal.

[14] Son M, Jo S, Lee JS, Lee DH (2020). Association between oral health and incidence of pneumonia: a population-based cohort study from Korea. Sci Rep.

[15] Yamguchi T, Mori K, Kojima Y (2024). Efficacy of perioperative oral care management in the prevention of surgical complications in 503 patients after pancreaticoduodenectomy for resectable malignant tumor: a multicenter retrospective analysis using propensity score matching. Surgery.

[16] Kubo A, Sakai K, Ueki S, Fujita K (2024). Effect of perioperative oral care on postoperative infections in patients with cancer: a systematic review and meta-analysis. Jpn J Nurs Sci.

[17] Moriwaki M, Toba M, Takizawa M (2025). Oral management improves patient outcomes in hematopoietic stem cell transplantation. Int Dent J.

[18] Yoneyama T, Yoshida M, Matsui T, Sasaki H (1999). Oral care and pneumonia. Lancet.

[19] Scannapieco FA (2006). Pneumonia in nonambulatory patients. The role of oral bacteria and oral hygiene. J Am Dent Assoc.

[20] Scannapieco FA, Bush RB, Paju S (2003). Associations between periodontal disease and risk for nosocomial bacterial pneumonia and chronic obstructive pulmonary disease. A systematic review. Ann Periodontol.

[21] Berger K, Schopohl D, Bollig A, Strobach D, Rieger C, Rublee D, Ostermann H (2018). Burden of oral mucositis: a systematic review and implications for future research. Oncol Res Treat.

[22] Chaudhry HM, Bruce AJ, Wolf RC (2016). The incidence and severity of oral mucositis among allogeneic hematopoietic stem cell transplantation patients: a systematic review. Biol Blood Marrow Transplant.

[23] Bellm LA, Epstein JB, Rose-Ped A, Martin P, Fuchs HJ (2000). Patient reports of complications of bone marrow transplantation. Support Care Cancer.

[24] Haverman TM, Raber-Durlacher JE, Rademacher WM (2014). Oral complications in hematopoietic stem cell recipients: the role of inflammation. Mediators Inflamm.

[25] Niscola P, Romani C, Cupelli L (2007). Mucositis in patients with hematologic malignancies: an overview. Haematologica.

[26] Furuya J, Suzuki H, Hidaka R (2022). Association between oral health and advisability of oral feeding in advanced cancer patients receiving palliative care: a cross-sectional study. Support Care Cancer.

[27] Shimizu A, Ohno T, Fujishima I, Kayashita J, Momosaki R, Nishioka S, Wakabayashi H (2023). Impact of poor oral health status on swallowing function improvement in older dysphagic patients. Cureus.

[28] Shiraishi A, Wakabayashi H, Yoshimura Y (2020). Oral management in rehabilitation medicine: oral frailty, oral sarcopenia, and hospital-associated oral problems. J Nutr Health Aging.

[29] Tattar R, da Costa BD, Neves VC (2025). The interrelationship between periodontal disease and systemic health. Br Dent J.

[30] Okubo R, Hoshi SL, Kondo M (2023). Cost-effectiveness of professional and mechanical oral care for preventing pneumonia in nursing home residents. J Am Geriatr Soc.

